# Collargol in Typhoid Fever

**Published:** 1909-08

**Authors:** 


### ABSTRACTS.

### COLLARGOL IN TYPHOID FEVER.

Dr. Th. Mironescu Medical Clinic of Bukarest University; *Berlin klin. Wochenschr.,* January 4, 1909), made a comparative test of treatment with collargol enemata and with cold baths in typhoid fever. The severity of the cases in which collargol was resorted to was evidenced by the temperature as well as the grave appearance of the patients. None the less, all seventeen collargol cases recovered, while there were four deaths in the seventeen others. The marked difference in the course of the disease was also strikingly evident. The collargol enemata always had a favorable effect on temperature and on general condition; the duration of the disease was shortened and complications were prevented. In the cases not treated with collargol the fever lasted ten to fifteen days longer. Intestinal hemorrhage never occurred in the collargol cases, while this complication was seen in one-third of the others; this fact was proved by examining the feces by Weber's method.

While typhoid fever is initiated by a leucopenia, collargol increased the leucocyte number to normal. Thus in one case with a leucocyte count of 4,000 the number rose to 7,000 in twenty-four hours after the first collargol enema. The leukocytosis is not without influence on the follicular intestinal ulcers, and this explains the non-occurrence of intestinal hemorrhage.

The rectal method enables the administration of very large doses of collargol. The patient gets daily one or two enemata of 75 grains collargol in 3½ ounces water preceded by a cleansing irrigation. When the enemata were not retained, 10 to 12 minims of tinct. opium were added to them; or if necessary 15-grain capsules of collargol (eight to ten per day) were used per os; but this is not so effective. The non-toxicity of collargol was shown by the fact that some of the patients took per rectum 150 grains daily, for ten to twelve days, without the least untoward symptom.

---

